# Functional Analysis of M-Locus Protein Kinase Revealed a Novel Regulatory Mechanism of Self-Incompatibility in *Brassica napus* L.

**DOI:** 10.3390/ijms20133303

**Published:** 2019-07-05

**Authors:** Fang Chen, Yong Yang, Bing Li, Zhiquan Liu, Fawad Khan, Tong Zhang, Guilong Zhou, Jinxing Tu, Jinxiong Shen, Bin Yi, Tingdong Fu, Cheng Dai, Chaozhi Ma

**Affiliations:** 1 National Key Laboratory of Crop Genetic Improvement, National Center of Rapeseed Improvement in Wuhan, Huazhong Agricultural University, Wuhan 430070, China; 2 Key Laboratory of Horticultural Plant Biology, Ministry of Education, Huazhong Agricultural University, Wuhan 430070, China

**Keywords:** *Brassica napus*, self-incompatibility, self-compatibility, *M* locus protein kinase (*MLPK*), RNAi, CRISPR/Cas9

## Abstract

Self-incompatibility (SI) is a widespread mechanism in angiosperms that prevents inbreeding by rejecting self-pollen. However, the regulation of the SI response in *Brassica napus* is not well understood. Here, we report that the M-locus protein kinase (*MLPK*) *BnaMLPKs*, the functional homolog of *BrMLPKs* in *Brassica rapa*, controls SI in *B. napus*. We identified four paralogue *MLPK* genes in *B. napus*, including *BnaA3.MLPK*, *BnaC3.MLPK*, *BnaA4.MLPK*, and *BnaC4.MLPK*. Two transcripts of *BnaA3.MLPK*, *BnaA3.MLPKf1* and *BnaA3.MLPKf2*, were generated by alternative splicing. Tissue expression pattern analysis demonstrated that *BnaA3.MLPK*, especially *BnaA3.MLPKf2*, is highly expressed in reproductive organs, particularly in stigmas. We subsequently created RNA-silencing lines and CRISPR/Cas9-induced quadruple mutants of *BnaMLPKs* in *B. napus* SI line S-70. Phenotypic analysis revealed that SI response is partially suppressed in RNA-silencing lines and is completely blocked in quadruple mutants. These results indicate the importance of *BnaMLPKs* in regulating the SI response of *B. napus*. We found that the expression of SI positive regulators S-locus receptor kinase (*SRK*) and Arm-Repeat Containing 1 (*ARC1*) are suppressed in *bnmlpk* mutant, whereas the self-compatibility (SC) element Glyoxalase I (*GLO1*) maintained a high expression level. Overall, our findings reveal a new regulatory mechanism of *MLPK* in the SI of *B. napus.*

## 1. Introduction

Self-incompatibility (SI) is an elaborate mechanism that promotes outcrossing and maintains genetic diversity in many flowering plants [1]. In the Brassicacease, SI is sporophytically regulated by a single genetic locus called the S-locus, at which multiple variants (now known as *S* haplotype) occur in any one species [2]. The S-locus encodes the stigma determinant of SI, the S-locus receptor kinase gene (*SRK*), which is a membrane-anchored Ser/Thr kinase localized in the plasma membrane of stigmatic papilla cells [3], and the pollen determinant of SI, a small secreted peptide localized in the pollen coat, which is known as S-locus cysteine-rich protein (*SCR*) [4] or the S-locus protein 11 (*SP11*) [5]. Biochemical studies have shown that SCR/SP11 is the ligand for SRK that are encoded in the same *S* haplotype [6,7]. This *S* haplotype-specific receptor–ligand interaction, and the resulting activation of a pollen-inhibitory signaling pathway upon self-pollination but not cross-pollination, explains the specificity of the SI response [8].

Some elements of SRK-mediated signaling have been reported in *Brassica* species. One report indicated that prior to self-incompatible pollination, SRK kinase activity is inhibited by the thioredoxin h proteins THL1 and THL2 [9]. Once self-incompatible pollen lands on the stigma, the interaction of the SRK extracellular domain with its pollen ligand is thought to separate the THL1 and THL2 proteins from SRK, allowing the activation of SRK and its downstream signaling [9]. Subsequently, the plasma-membrane-tethered M-locus protein kinase (MLPK) is thought to interact with the activated SRK [10]. Another SRK interactor is the arm-repeat-containing protein ARC1, an E3 ubiquitin ligase [11], that can ubiquitinate Exo70A1 and direct this putative component of the exocyst for degradation by the proteasome [12]. Degradation of Exo70A1 is thought to inhibit the exocytosis of multivesicular bodies, presumably precluding the release of factors required for successful pollen tube growth (compatibility factors) and causing rejection of self-pollen [13]. ARC1 was reported to cause the degradation of at least one compatibility factor, glyoxalase I (GLO1) [14], a protein that is required for the detoxication of methylglyoxal, the cytotoxic by-product of glycolysis [15]. So far, the involvements of THL1/2, MLPK, ARC1, and Exo70A1 in SI have been reported only in a subset of *Brassica* species. However, their roles remain controversial [16,17] given studies in transgenic *Arabidopsis thaliana* self-incompatible *SRK/SCR* plants that found no evidence for the proposed roles of these genes in SI [16,18].

In this study, we examined the role of *MLPK* in the SI response of *Brassica napus*. *MLPK* was originally identified by positional cloning using F_2_ populations derived from a cross between a self-incompatible *Brassica rapa* S_8_ homozygote (*S_8_S_8_MM*) and a self-compatible *B. rapa* mutant Yellow Sarson C634 (*S_f2_S_f2_mm*) [19]. The ability to reject self-pollen was restored in mutant papilla cells by transient expression of wild-type *BrMLPK* in these cells, and bimolecular fluorescence complementation (BiFC) experiments indicated that BrMLPK interacts with SRK in vitro [10]. These results suggest that *BrMLPK* functions as a positive self-incompatibility factor in the SI signaling pathway of *B. rapa*. However, several questions regarding the role of *MLPK* remain unresolved in SI. First, this role has not been confirmed in stably-transformed Yellow Sarson mutant plants. Second, functional analysis of *MLPK* has only been performed in *B. rapa*. Third, *AtAPK1b*, the *A. thaliana* gene that shares the highest sequence identity with *BrMLPK*, was reported to play no role in the SI response of self-incompatible transgenic *A. thaliana* [20]. Thus, the function of *MLPK* may vary among Brassicaceae species and how this protein might be involved in the SI response of the allotetraploid *B. napus* is still unknown.

To resolve these issues, we used a homology-based candidate gene approach to clone *BnaMLPK* sequences from a self-incompatible *B. napus* strain. Here, we report the identification and analysis of the four paralogues of *BrMLPK* in *B. napus*. We show that transgenic plants generated by RNA interference (RNAi)-based silencing and CRISPR/Cas9-based gene editing of *BnaMLPKs* exhibit partial and complete breakdown of SI, respectively. We show that this breakdown is associated with a drastic reduction of *SRK* transcripts in mutant stigmas. Our results reveal a novel function of *BnaMLPK* as a positive regulator of the expression of SI-relevant genes in Brassicaceae stigmas.

## 2. Results

### 2.1. Cloning and Sequence Analysis of BnaMLPKs from Self-Incompatibile B. napus

To investigate the function of *MLPK* in self-incompatible *B. napus*, the genome sequence of *MLPK* was obtained from the *Damor-bzh* genome database [21] using *BrMLPK* as the reference sequence. *B.napus* possesses four *MLPK* genes: *BnaA3.MLPK*, *BnaC3.MLPK*, *BnaA4.MLPK*, and *BnaC4.MLPK*. Then, the *MLPK* sequences from self-incompatible *B. napus* S-70 were cloned by PCR based on *Damor-bzh MLPK* sequences. The genome sequence of different paralogue genes ranged from 2080 to 2288 base pairs (bp), and the CDS sequence ranged from 1179 to 1306 bp (Table S1). *BnaA3.MLPK* and *BnaC3.MLPK* contain six exons and five introns. *BnaA4.MLPK* and *BnaC4.MLPK* contain five exons and four introns. The sequence identification of the four MPLK proteins and nucleotides is very high (Table 1; Figure S1). BnaA3.MLPK, BnaC3.MLPK, BnaA4.MLPK, and BnaC4.MLPK are closer to BrMLPKf1/2, BoMLPKf1, BoMLPKn1, and BoMLPKn1, respectively (Table 2; Figure S1).

### 2.2. Sequence Analysis of BnaMLPK Transcripts

**Figure 1:**
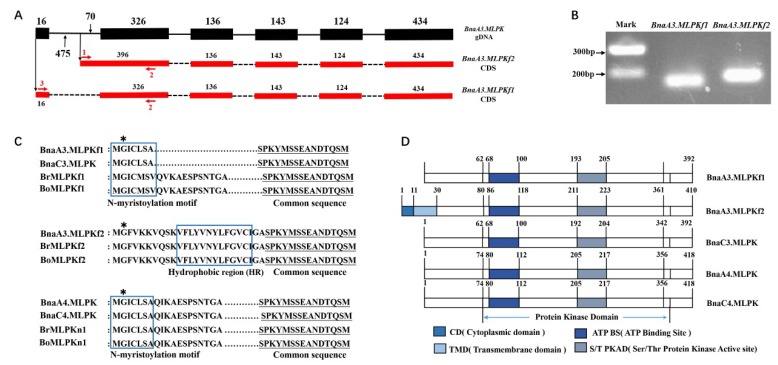
Comparison of the N-terminal amino acid sequences of MLPK in *Brassica*. (**A**) Schematic diagram of nucleotide sequences of the alternative transcription region of the *BnaA3.MLPK* gene. The black numbers indicate the base numbers. Red numbers and arrows mean the positions of primers used in (**B**). (**B**) The electrophoretogram of *BnaA3.MLPKf1* and *BnaA3.MLPKf2* transcripts. (**C**) Comparison of the deduced amino acid sequences of MLPK in *Brassica*. The blue boxes indicate the predicted myristylation consensus sequences in BnaA3.MLPKf1, BnaC3.MLPK, BrMLPKf1, BoMLPKf1, BnaC4.MLPKn1, BnaA4.MLPK, BrMLPKn1, and BoMLPKn1 and the specific hydrophobic regions in BnaA3.MLPKf2, BrMLPKf2, and BoMLPKf2. The common sequences are underlined. The conserved second-position Gly residues are indicated by asterisks. (**D**) Protein secondary structure prediction of BnaMLPKs.

Previous studies indicated that two isoforms of *BrMLPK* and *BoMLPK* are generated by alternative splicing [10,22]. We wondered if the alternative splicing still exists in *BnaMLPKs*. To confirm this hypothesis, we cloned the transcript sequences of *BnaMLPK* in S-70 using primers designed based on two isoforms of *BrMLPK* (*BrMLPKf1* and *BrMLPKf2*) sequences (Table S2). Two different sequences were detected by Sanger sequencing (Table S1), which were transcribed by *BnaA3.MLPK* (Table S1; Figure 1A), named *BnaA3.MLPKf1* and *BnaA3.MLPKf2*. *BnaA3.MLPKf1* starts to transcribe from the first exon to the last exon*,* whereas the transcriptome of *BnaA3.MLPKf2* initiates at the 475th base of the first intron (Figure 1A). The sequence length of *BnaMLPKf2* transcript is 54 bp longer than that of *BnaMLPLf1*, which was detected by PCR (Figure 1B). However, other MLPK paralogues contain one transcript. Therefore, a total of five transcripts were obtained from the four *BnaMLPK* genes. The domain analysis result indicated that, except for BnaA3.MLPKf2, the other four BnaMLPK proteins contain a typical plant N-myristylation consensus sequence at the N-terminal [23,24] (Figure 1C). The C-terminal of these five proteins is conserved, which contains a protein kinase domain (Figure 1D).

### 2.3. Phylogenetic and Tissue-Specific Expression Analysis of BnaMLPKs

**Figure 2:**
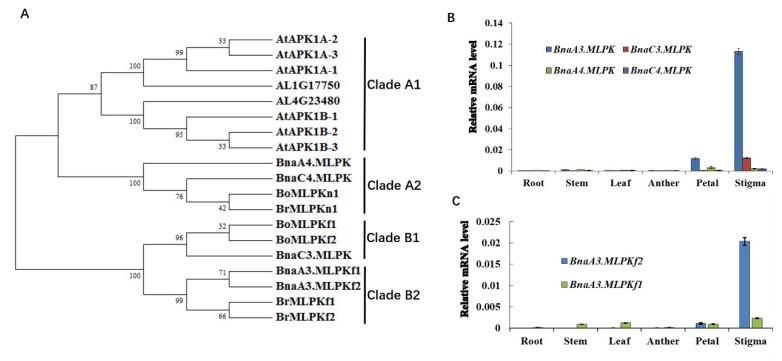
Phylogenetic and tissue-specific expression analysis of *BnaMLPK* isoforms. (**A**) The phylogenetic tree was analyzed using the neighbor-joining (NJ) algorithm using predicted MLPK amino acid sequences from *B. napus*, *Brassica rapa*, *Brassica oleracea*, *Arabidopsis thaliana*, and *Arabidopsis lyrata*. Confidence values from the bootstrap test (1000 replicates) are marked by the numbers on the tree. Clade A1, Clade A2, Clade B1, and Clade B2 indicate four sub-clades. BrMLPKf1, BrMLPKf2, and BrMLPKn1 are the proteins in *B. rapa*; AtAPK1A-1, AtAPK1A-2, AtAPK1A-3, AtAPK1B-1, AtAPK1B-2, and AtAPK1B-3 are the homologous proteins of BrMLPKs in *A. thaliana*; AL1G17750 and AL4G23480 are the homologous proteins of BrMLPKs in *A. lyrata*; BoMLPKf1, BoMLPKf2, and BoMLPKn1 are the homologous proteins of BrMLPKs in *B. oleracea*; and BnaA3.MLPKf1, BnaA3.MLPKf2, BnaC3.MLPK, BnaA4.MLPK, and BnaC4.MLPK are cloned homologous genes of BrMLPKs in *B. napus*. (**B**) Tissue-specific expression of the *BnaA3.MLPK*, *BnaC3.MLPK*, *BnaA4.MLPK*, and *BnaC4.MLPK* in different tissues of *B. napus*. The expression levels of each transcript were detected by real-time quantitative reverse transcription PCR (qPCR). The *actin* gene was used as control. (**C**) Tissue-specific expression of the two isoforms *BnaA3.MLPKf1* and *BnaA3.MLPKf2* in different tissues of *B. napus*.

We explored the evolutionary relationship of MLPK proteins from various species using phylogenetic analysis. Four major clades, Clade A1, Clade A2, Clade B1, and Clade B2, were obtained (Figure 2A). BnaA3.MLPKf1 and BnaA3.MLPKf2 were in Clade B2, and BnaC3.MLPK was in Clade B1 (Figure 2A). BnaA4.MLPK and BnaC4.MLPK were in Clade A2 (Figure 2A). Then, quantitative real-time PCR (qRT-PCR) was performed to analyze the expression pattern of *BnaMLPKs* in different tissue. Both *BnaA3.MLPK* and *BnaC3.MLPK* are enriched in the stigma, but the expression of *BnaA3.MLPK* is much higher than *BnaC3.MLPK* (Figure 2B). The expressions of *BnaA4.MLPK* and *BnaC4.MLPK* are very low in all tissues (Figure 2B). Then, we tested different isoforms of *BnaA3.MLPK* in different tissues. The results indicate *BnaA3.MLPKf2* is enriched in the stigma and petal, which is much higher than *BnaA3.MLPKf1* (Figure 2C). These results demonstrate that *BnaA3.MLPK* and *BnaC3.MLPK* might participate in the regulation of SI responses, and *BnaA3.MLPKf2* is the major isoform expressed in the stigma.

### 2.4. RNAi Knockdown of BnaMLPKs Partially Suppressed SI Response in B. napus

**Figure 3:**
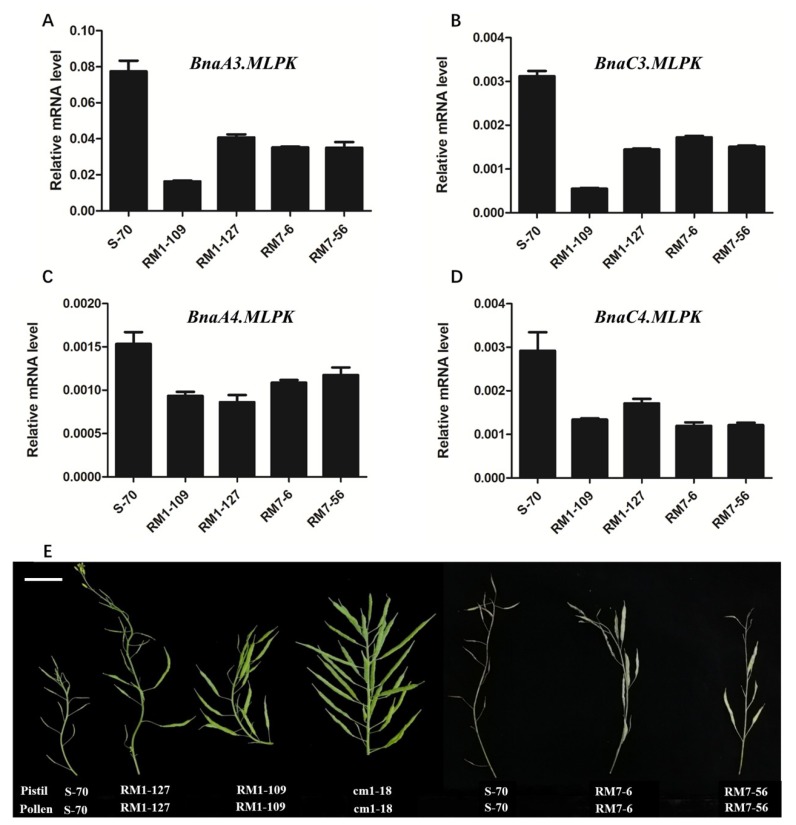
RNAi experiment demonstrated that *MLPK* is required for incompatible pollination. (**A**–**D**) The expression analysis of *BnaMLPKs* in hpRNAi transgenic lines. The total RNA was extracted from at least thirty stigmas for each pollination treatment. Data represent the average of three technical replicates (±SE). Similar expression results were acquired with three biological replicates. The *actin* gene was considered as the control reference. RM1-109 and RM1-127 indicate the two different positive plants from the RM1 hpRNAi construct. RM7-6 and RM7-56 denote the two transgenic plants from the RM7 hpRNAi construct. All four genes of *MLPK* were down-regulated in un-pollinated stigmas of RM1 and RM7 transgenic lines. The un-pollinated wild type S-70 was used as control. (**E**) Images of mature siliques from pistils of S-70, RM1-127, RM1-109, cm1-18, RM7-6, and RM7-56 lines following self-pollination. S-70 and cm1-18 were used as controls. Scale bar = 1 cm.

Though *MLPK* is a positive regulator of the SI response in *B. rapa*, the specific roles in *B. napus* remain unclear. To validate the function of *BnaMLPKs* in S-70 *B. napus*, we attempted to generate RNAi transgenic lines of *BnaMLPKs* using the stigma-specific *SLR1* promoter to drive the expression of two hairpin RNA interference (hpRNAi) constructs [25], which were used to suppress the expression of *BnaA3.MLPK/BnaC3.MLPK* (RM1) and *BnaA4.MLPK /BnaC4.MLPK* (RM7) (Figure S2). The expressions of all four *BnaMLPKs* were partially suppressed in four RNAi transgenic lines, ranging from 25% to 60% (Figure 3A–D). Phenotypic analysis showed that the seed setting was partially rescued in *bnmlpk–RNAi* lines (Figure 3E), indicating that *BnaMLPKs* positively regulate SI response.

### 2.5. Knockout Mutant of BnaMLPKs Created Using CRISPR/Cas9 System Completely Breaks Down SI Response in B. napus

The SI response was partially suppressed by knock down expression of *BnaMLPKs* in RNAi lines, but which paralogue is the dominant gene remained unclear. To address this question, two sgRNAs were designed to target the conserved sequences among the four paralogous genes (Figure S3A,B). By genetic transformation into S-70, we obtained six positive plants that were further confirmed by PCR using *Cas9*-specific primers (Figure S3C). Next, Sanger sequencing was used to assess the editing efficiency. The four *BnaMLPK* genes were examined in the six transgenic plants. As a result, the mutation frequency at the sgRNA1 target site ranged from 83.3% to 100% for each of the four genes, and at sgRNA2 target site, ranged from 66.7% to 100.0% (Table 3). Among all types of mutations, 16.7% (4/24) were deletions, 12.5% (3/24) were insertions, and 13.9% (17/24) were combined mutations (Figure S4).

Both alleles for each gene might be mutated by CRISPR/Cas9, which could produce five genotypes: homozygote, bi-allele, heterozygote, chimera, and WT. To estimate the proportion of each genotype among the transgenic lines, we detected the mutation type of each targeted site using sequencing. All amplicons were analyzed by inserting them into a TA vector and sequencing 10 individual clones for each of the 48 amplicons. The genotype data are summarized in Table 4. The genotyping results showed that 10.4% (5/48) sites were homozygous and 43.8% (21/48) sites were bi-allelic. Thus, a total of 54.2% sites had defects in both alleles (Table 4). The frequencies of heterozygotes and chimeras were 10.4% (5/48) and 25.0% (12/48), respectively. No mutations were found in 8.3% (4/48) of the sites.

**Figure 4:**
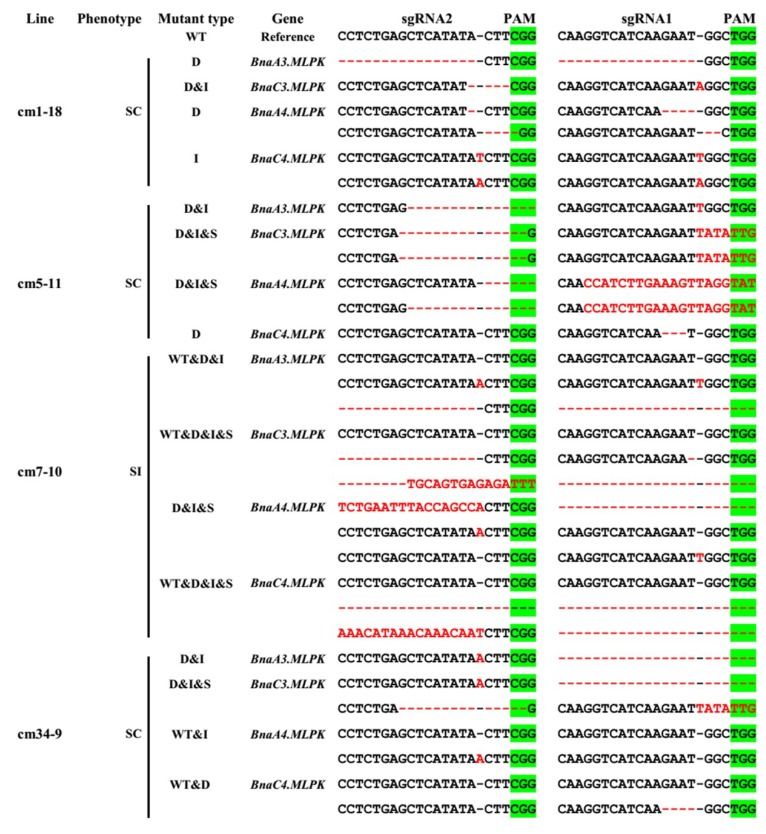
The editing information of *BnaMLPKs* in T_1_ generation mutants and sequencing analysis of the four T_1_ generation lines (named cm1-18, cm5-11, cm7-10, and cm34-9). The DNA was extracted from the leaves. Representative sequences of the mutated *MLPK* were aligned with those of the reference gene. The protospacer adjacent motif (PAM) region is marked by green. The two sgRNAs are sgRNA1 and sgRNA2. The inconsistent region indicates that the sequence is edited, which is denoted by red. SI: self-incompatibility; SC: self-compatibility; D: deletion; I: insertion; S: substitution; WT: wild-type S-70 sequence.

**Figure 5:**
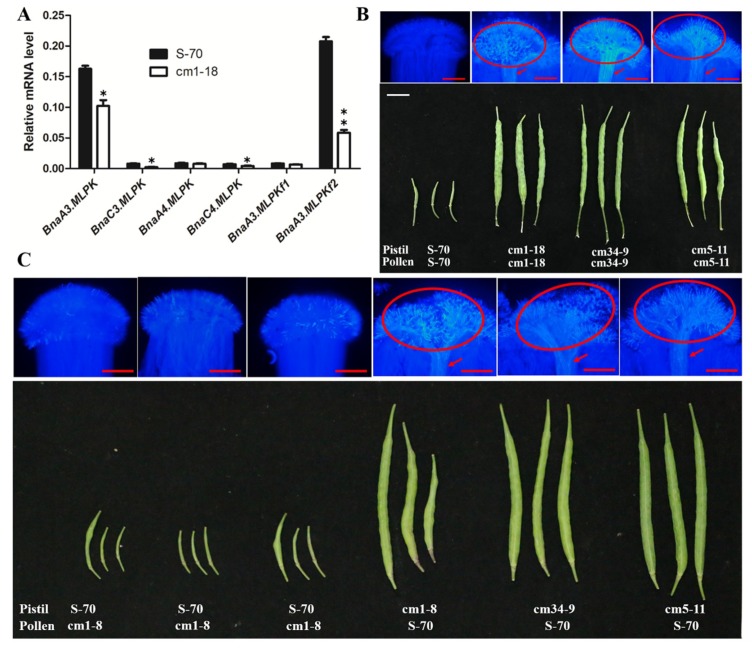
*MLPK* is required for incompatible pollination in *B. napus* (**A**) Expression analysis of *BnaMLPKs* in *bnamlpk* mutant. The total RNA was extracted collectively from at least 30 stigmas for each pollination treatment. Data represent the average of three technical replicates (±SE). Similar expression results were acquired with three biological replicates. *Actin* gene was used as the control reference. All four genes of *MLPK* were down-regulated in un-pollinated stigmas of *bnamlpk* mutants. The un-pollinated wild-type S-70 was used as the control. The asterisks represent significant differences (ANOVA: * *p* < 0.05, ** *p* < 0.01). The black bar indicates the stigmas of un-pollinated wild-type S-70. The white bar represents the stigmas of un-pollinated mutant cm1-18. (**B**) The upper plate indicates aniline blue staining. The aniline blue assays were performed 16 h after self-pollination. Red arrows indicate the pollen tubes. The red circles indicate germinated pollen grains. Scale bar = 50 µm. The lower plate represents mature siliques developed from pistils of S-70, cm1-18, cm34-9, and cm5-11 lines following pollination as indicated. Scale bar = 1 cm. (**C**) The upper plate indicates aniline blue staining. The aniline blue assays were performed 16 h after different pollination treatments as indicated in the lower plate. Red arrows show the pollen tubes. The red circles indicate germinated pollen grains. Scale bar = 75 µm. The lower plate represents mature siliques developed from pistils of S-70, cm1-18, cm34-9, and cm5-11 lines following pollination as indicated. Scale bar = 1.5 cm.

The six positive transgenic plants were self-crossed to obtain the T_1_ population. Mutations of *BnaMLPKs* at the sgRNA1 and sgRNA2 target sites were examined in four plants (cm1, cm5, cm7, and cm34) from T_1_ lines. Among the transgenic plants, cm1-18 and cm5-11 lines were quadruple mutants of *BnaMLPKs* without wild-type sequence (Figure 4; Table 4). New mutation types were identified in the T1 generation of cm5. One possible reason is that Cas9 could be still functional at the non-mutated allele at the targeted region. Then, all the *BnaMLPK* genes showed down-regulated expression in the cm1-18 line compared with the wild-type S-70, especially *BnaA3.MLPK*, *BnaA3.MLPKf2*, *BnaC3.MLPK*, and *BnaC4.MLPK* (Figure 5A). Compared to the SI line S-70, abundant pollen germination, pollen tube elongation, and many seeds setting were observed in quadruple *bnamlpk* mutant after self-pollination (Figure 5B). When *bnamlpk* pollen was pollinated to S-70 stigmas, no pollen germination and seed setting were observed (Figure 5C), suggesting that *BnaMLPKs* did not change the pollen SI determination. These results demonstrate that *BnaMLPK* is a positive regulator of the SI response in *B. napus*.

### 2.6. Expression of SI-Related Genes Changed in bnamlpk Mutant

**Figure 6:**
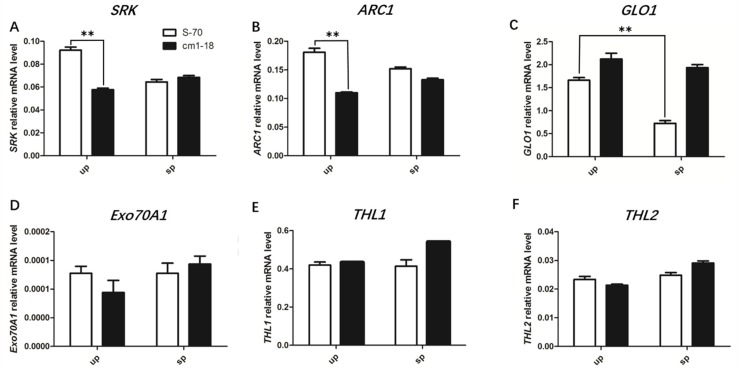
Quantitative real-time PCR analysis of the expression pattern of (**A**) *SRK*, (**B**) *ARC1*, (**C**) *GLO1*, (**D**) *Exo70A1*, (**E**) *THL1,* and (**F**) *THL2* genes in the stigmas of wild-type and mutant *B. napus* S-70. The total RNA was extracted collectively from at least 30 stigmas for each treatment. Data represent the average of three technical replicates (±SE). Similar expression results were acquired with three biological replicates. The *actin* gene was used as the control reference. Asterisks indicate significant differences (ANOVA: **p < 0.01). The white bar indicates wild-type S-70 stigmas of un-pollination (up) or self-pollination (sp) and the black bar denotes the mutant cm1-18 stigmas of un-pollination (up) or self-pollination (sp).

Previous studies reported that *SRK*, *ARC1*, *THL1/2*, *Exo70A1* and *GLO1* genes are mainly involved in the SI of *B. napus* [3,9,11,12,14,26]. So, we wanted to know whether the SC phenotype of *bnamlpk* is caused by changing the expression of SI-related genes. qRT-PCR was used to detect the relative expression levels of these genes in the stigmas of *bnamlpk* mutant and wild type (S-70) after un-pollination (up) and self-pollination (sp). Without pollination, *SRK* and *ARC1* were suppressed in *bnamlpk* compared to the S-70 (Figure 6A,B). After self-pollination, these two genes were down-regulated (*SRK*: ~1.4 fold, *ARC1*: ~1.2 fold) in S-70, but not in *bnamlpk* (Figure 6A,B). The expression of *GLO1* significantly decreased (~2.3 fold) in S-70 after self-pollination (Figure 6C). *GLO1* was slightly up-regulated (~1.3 fold) in *bnamlpk* without self-pollination (Figure 6C). The expression of the other genes showed almost no differences between the mutant and wild-type with or without self-pollination (Figure 6D–F). These results suggest that *BnaMLPKs* might regulate the expression of *SRK*, *ARC1*, and *GLO1* to control the SI response in *B. napus*.

## 3. Discussion

### 3.1. BnaMLPK is a Positive Regulator of SI Response in B. napus

SRK-mediated signaling has been reported in *Brassica* species [13,27,28,29]. MLPK, a plasma membrane-tethered M-locus protein kinase, is phosphorylated by SRK and required for the SI signaling pathway in *B. rapa* [10,19]. However, the homolog of *BrMLPK* in *Arabidopsis* plays no role in the SI response in self-incompatible transgenic *A. thaliana* (*SRKb-SCRb*) [20]. Therefore, more evidence is required to clarify the roles of *MLPK* in the SI response of Brassicacea. In this study, knock-down and knock-out of *BnaMLPKs* was performed in self-compatible *B. napus* S-70, which broke down the SI response, suggesting that *BnaMLPKs* are positive regulators of the SI response.

The sgRNA that targeted the conserved sequences among the four paralogous genes in the CRISPR/Cas9 system could in theory generate single, double, triple, and quadruple mutants. However, no single mutant *bnamlpk* line was separated in the present transgenic materials, probably due to the high genome editing efficiency. The cm34-9 line showed a SC phenotype (Figure 4), the genotypes of *BnaA3.MLPK* and *BnaC3.MLPK* were homozygous mutations, and *BnaA4.MLPK* and *BnaC4.MLPK* were heterozygous mutations. Based on the expression pattern and phenotype of the mutant, we propose that *BnaA3.MLPK* and *BnaC3.MLPK* are the functional genes of SI in *B. napus*. However, which paralogue gene plays the dominant function in the SI response remains unknown. The single mutants of *BnaA3.MLPK* and *BnaC3.MLPK* could be isolated in the next generation, which would help us to answer this question.

### 3.2. Alternative Splicing Involved in MLPK-Regulated SI in B. napus

Alternative splicing (AS) of eukaryotic transcripts is a mechanism that enables cells to generate vast protein diversities from a limited number of genes. Studies in several plants have indicated that tissue-specific AS mediates tissue differentiation and promotes specialized characteristics [30]. Alternative splicing at first exons (AFEs) has been well studied in mammals, which is suggested to contribute to the diversification of gene expression [31]. Transcript analysis showed that two isoforms of *BnaA3.MLPK* are generated by AFEs. The expression patterns of these two isoforms of *BnaA3.MLPK* are significantly different, indicating the regulation of AFEs in gene expression.

*B. napus* (AACC, 2*n* = 38), as an allotetraploid species, was developed by natural allopolyploidization between *B. rapa* (AA, 2*n* = 20) and *B. oleracea* (CC, 2*n* = 18) [21]. *MLPK* plays an important role in the SI response of *B. rapa* [10], but not in transgenic *A. thaliana* (*SRKb-SCRb*) [10]. These results imply that the function of *MLPK* has been differentiated between *Arabidopsis* and *Brassica* during evolution [32]. *BrMLPK* was found to generate two transcripts, *BrMLPLf1* and *BrMLPKf2*, by AFEs, both of which function in SI signal transduction in the stigma of *B. rapa* [10]. This study also demonstrates that *BnaA3.MLPKf1* and *BnaA3.MLPKf2* are the major isoforms of *BnaA3.MLPK* in *B. napus*. Further experiments are need to determine the functions of the two transcripts, such as a complementary experiment transforming two *BnaA3.MLPK* transcripts into the *bnamlpk* mutant by rescuing the SI phenotype.

### 3.3. BnaMLPKs Regulate SI Responses by Influencing the Expression of SCR-SRK Pathway Components

Several genes have been identified in the SRK-SCR pathway [33]. Although the regulatory mechanism of these genes is well-studied at the protein level, little has been reported about their regulatory mechanism at the transcription levels. We found the expressions of *SRK* and *ARC1* were suppressed in *bnamlpk* before self-pollination (Figure 6A,B). Down-regulated expressions of *SRK* and *ARC1* prevented the SI signal transduction, resulting in pollen tubes growth and elongation. Without pollination, the self-compatible factor *GLO1* was slightly induced in *bnamlpk*. These results imply that the inhibition of *SRK* and *ARC1* and the maintained high level of *GLO1* cause the breaking down of the SI response in *bnamlpk*. The mechanism through which MLPK regulates the expressions of *SRK*, *ARC1*, and *GLO1* needs to be further investigated.

Given the similar sequences, expression patterns, and transcriptional processing of *BnaMLPK* and its *B. rapa* homologue *BrMLPK*, the expectation is that the BnaMLPK protein would be required for the SI response of *B. napus* and would have the features and function previously reported for the BrMLPK protein, i.e., plasma-membrane localization, interaction with the SRK receptor, and involvement in SRK-mediated signaling. Consistent with previous reports, we found that down-regulation or disruption of *BnaMLPK* caused partial or complete loss of SI, thus confirming a role of *BnaMLPK* in SI. However, our results show that the self-compatibility phenotype of *bnmlpk* mutants is associated with a drastic reduction in the level of *SRK* transcripts in un-pollinated stigmas, a reduction that is known to cause breakdown of SI [34,35]. Although we did not investigate the signaling role of the BnaMLPK protein, we did reveal a novel role of BnaMLPK that had not been reported for BrMLPK. BnaMLPK may act independently of SRK signaling to regulate the transcript levels of genes required for SI. Further analysis is required to understand how this SRK-independent signaling regulatory function is achieved and if it involves transcriptional control in the nucleus by as-yet-unknown transcription factors or post-transcriptional control of transcript stability in the cytoplasm.

## 4. Materials and Methods

### 4.1. Plant Materials and Growth Condition

The self-incompatible *B. napus* S-70 species was separated from the self-incompatible S-1300, which was acquired by interspecific hybridization between the self-compatible *B. napus* line Huayou8 and the self-incompatible *B. rapa* Xishuibai [36]. S-70 and its relative transgenic lines were grown in the transgenic field of Huazhong Agriculture University, Wuhan, China, during *B. napus* growing season, or cultivated in a growth room under the light intensity of 100 μmol·m^−2^s^−1^ with a 16/8 h light/dark photoperiod at 22 °C.

### 4.2. Sequence Cloning of BnaMLPKs

To clone the genome DNA (gDNA) or CDS region of *MLPKs*, the *B. napus* genomic DNA or total RNA was extracted from leaves or stigmas following the CTAB method [37] or plant mini RNeasy kit (Qiagen, Hilden, Germany) according to the manufacturer’s protocol. Approximately 1 μg of total RNA was used for cDNA synthesis with the Thermo Scientific Revertaid First Strand cDNA Synthesis kit (Thermo, New York, USA). Primers M4CDS-F/M4CDS-R were used to amplify the four paralogues of the *MLPK* genes (Table S2), followed by standard PCR procedure: 98 °C for 2 min; 98 °C for 20 s, 55 °C for 20 s, 72 °C for 1.5 min, 35 cycles; 72 °C for 10 min; and 25 °C for 5 min. Two primer pairs (Table S2), BrMLPKf1-F/BrMLPKf1-R and BrMLPKf2-F/BrMLPKf2-R, were designed according to the *BrMLPKf1/2* cDNA sequence [10,22], which were used to amplify the transcripts of *BnaMLPK*. PCR was performed using the following procedure: 98 °C for 2 min; 98 °C for 20 s, 55 °C for 20 s, 72 °C for 1 min, 35 cycles; 72 °C for 10 min; and 25 °C for 5 min. All PCR products were purified and ligated into the pMD18-T vector (Takara, Wuhan, China). Subsequently, the positive clones were confirmed via sequencing (TsingKe, Wuhan, China).

### 4.3. Tissue-Specific Expression Analysis of BanMLPKs

Total RNA from different tissues was extracted using a Plant Total RNA Isolation Kit (Sangon Biotech, Shanghai, China, No. SK8631) following the manufacturer’s instructions. Approximately 1 μg of total RNA was used for cDNA synthesis using a PrimeScriptTM RT reagent kit (TaKaRa, Tokyo, Japan, Cat#RR047A). A 10 μL total volume of reaction mixture was used for qPCR, which contained 5 μL 2× SYBR Green master mix (Achard et al., 172-5124, BioRad), 0.5 μL 5× diluted cDNA, 0.25 μL of each primer, and 4 μL ddH_2_O. Amplification was performed using a CFX Connect™ system (Bio-rad, USA). The amplification program involved one cycle of 95 °C for 5 min, followed by 50 cycles of 95 °C for 15 s, 60 °C for 20 s, and 72 °C for 20 s. The fluorescent products were detected at the third step of each cycle. The expression level of each gene was calculated using the 2^−ΔΔCT^ method. All analyses were repeated with three biological replicates. The *actin* gene (Gene-Bank accession no: AF111812) served as the internal control. All primers are listed in Table S2.

### 4.4. Phylogenetic Analysis

The genomic, full coding DNA sequence (CDS), and protein sequences of four paralogue *Brassica napus* MLPKs were aligned by DNAMAN (version5.2.2, Lynnon BioSoft, USA, https://www.lynnon.com/pc/framepc.html).

For phylogenetic analysis, homolog sequences MLPK in *A. thaliana* and *A. lyrata* were obtained from the TAIR Web site (https://www.arabidopsis.org/). The BrMLPK and BoMLPK protein sequences were used as previously reported [22]. The BnaMLPK protein sequences were obtained from DNAMAN-predicted CDS sequence. The sequence alignment was performed using Clustal Omega (http://www.ebi.ac.uk/Tools/msa/clustalo). An unrooted phylogenetic tree was constructed using MEGA7 (http://www.megasoftware.net), and aligned with default parameters (gap opening penalty = 10, gap extension penalty = 0.1), the neighbor-joining (NJ) statistical method, bootstrap analysis (1000 replicates), and pairwise gap deletion mode.

### 4.5. Plasmid Construction and B. napus Transformation

The *GENE-sgRNA* plant expression vectors were constructed following a previously reported method with minor modifications [38]. The target sgRNA sequences were designed using the web server CRISPR-P (http://cbi.hzau.edu.cn/cgi-bin/CRISPR;). Using *pCBC-DT1T2* as the template, two *AtU6 promoter-sgRNA-AtU6 terminator* cassettes were amplified by PCR using the primers listed in Table S2. The PCR fragments were inserted into *pKSE401* by Golden Gate Assembly [39], and confirmed by Sanger sequencing. These vectors were then used for plant transformation.

The hairpin RNA interference (hpRNAi) construct was reformed from the pCAMBIA2300 vector. The reformed construct contains a stigma-specific *SLR1* promoter [40], an intron, and a fragment of poly A (Figure S2). The sense fragment with nucleotides 908 to 1107 from the coding sequence of *BnaA3.MLPK* (CDS_908–1107_), including KpnI at N-terminal and SacI at the C-terminal, was amplified and inserted between the intron and the poly A. Then, the reverse complement fragment of CDS_908–1107_ with PstI at the N-terminal and XbaI at the C-terminal was developed and inserted between the *SLR1* promoter and the intron. Similarly, the sense fragment with nucleotides 1007 to 1206 from the coding sequence of *BnaC4.MLPK* (CDS_1007–1206_), including KpnI at the N-terminal and SacI at theC-terminal, was amplified and inserted between the intron and the poly A. Then, the reverse complement fragment of CDS_1007–1206_ with PstI at the N-terminal and XbaI at the C-terminal was developed and inserted between the *SLR1* promoter and the intron. The reconstructed vector was transformed into *Agrobacterium* GV3103 for plant transformation.

The *Agrobacterium*-mediated transformation was completed [41]. The explants were incubated in the *Agrobacterium*-infection buffer (MS, 4.43 g·L^−1^; sucrose, 30 g·L^−1^; acetosyringone, 100 mM·L^−1^; pH 5.8–5.9) for 20 min, and then transferred to M1 medium plates (MS, 4.43 g·L^−1^; sucrose, 30 g·L^−1^; acetosyringone, 100 mM·L^−1^; mannitol, 18 g· L^−1^; 2,4-D, 1 mg·L^−1^; kinetin, 0.3 mg·mL^−1^; pH, 5.8–5.9). The explants were kept in dark for 48 h. Afterward, the explants were transferred to M2 medium plates (MS, 4.43 g·L^−1^; sucrose, 30 g·L^−1^; acetosyringone, 100 mM·L^−1^; mannitol, 18 g·L^−1^; AgNO_3,_ 4 mg·L^−1^; 2,4-D, 1 mg·L^−1^; kinetin, 0.3 mg·mL^−1^; Timentin, 270 mg·L^−1^; pH, 5.8–5.9) with appropriate antibiotics to induce callus growth. The calluses were transferred to M3 (MS, 4.43 g·L^−1^; glucose, 10 g·L^−1^; xylose, 0.25 g·L^−1^; zeatin, 2 mg·L^−1^; IAA, 0.1 mg L^−1^; Timentin, 270 mg·L^−1^; pH, 5.8–5.9), followed by culturing in M4 medium (MS, 2.22 g·L^−1^; sucrose, 10 g·L^−1^; IBA, 0.5 mg L^−1^; Timentin, 135 mg·L^−1^; pH, 5.8–5.9) to allow the regeneration of shoots and roots.

### 4.6. Mutant Screening and Validation of Genome Editing

To analyze the mutations caused by CRISPR/Cas9, genomic DNA was extracted from leaves using the CTAB method [37]. The flanking sequences of the CRISPR target sites were amplified by PCR using gene-specific primers (Table S2). Then, most of the amplicons were directly sequenced. To decode mutations, the online tool DSDecode (http://dsdecode.scgene.com/;) was used for chromatogram decoding. The sequences files and the reference gene sequences were uploaded to the server and analyzed using default settings. The results were aligned with the reference sequences to ensure that the mutations were in the sgRNA targeted sites. For complex mutations, the amplicons were first sub-cloned into the *pGEM18-T* vector (Cat#A3610, Takara, Tokyo, Japan), and about 10 clones for each amplicon were individually sequenced. To analyze the RNAi lines, we used specific primers to detect the positive lines including *SLR1-RNAi* function fragment (Table S2). All the transgenic plants, including CRISPR/Cas9 mutants, were transferred to soil for further analysis.

### 4.7. Aniline Blue Staining Assay

The aniline blue staining was performed as reported with minor modifications [42]. After 16 h of pollination, the pistils were collected from the flowers, and immediately fixed in 3:1 ethanol/ glacial acetic acid for 2 h. The samples were further softened with 1 M NaOH at 60 °C for 1.5 h and then washed three times with distilled water. Pollen tubes were then stained by 0.1% decolorized aniline blue (pH9–11, in 0.1 M K_3_PO_4_) and placed in the dark for 6 h. The stained samples were observed under a fluorescence microscope (Ax10, Zeiss, Berlin, Germany) equipped with a UV filter set.

### 4.8. Self-Incompatibility Assay

Self-incompatibility phenotypic observation was performed [36]. At the flowering stage, the major inflorescence and two or three secondary ramifications were bagged for self-pollination after removing all blooming flowers artificially. The bags were shaken gently every two days to ensure sufficient self-pollination. The transgenic or wild-type flowers were cross pollinated after emasculation one day before anthesis. After two weeks, the bags were removed to allow the growth of seeds. The phenotypes were observed after maturation of seeds.

## 5. Conclusions

In this study, we cloned the *BrMLPK* homologous gene in *B. napus*, analyzed the transcripts in stigmas, and revealed their expression patterns in different tissues. We also demonstrated that *BnaMLPKs* positively regulate the SI response in the *B. napus*. Combined with the results from previous SI studies, we speculate that MLPK mediates the SI signaling pathway through regulating the expression of *SRK*, *ARC1*, and *GLO1*. These findings not only strengthen our understanding of the molecular roles of MLPK in the SI response of *B. napus*, but also directly contribute to the future study of the molecular mechanisms of SI in *Brassica*.

## Figures and Tables

**Table 1:** Sequence analysis of CDS and proteins of *MLPKs* in *Brassica napus.*

Gene	*BnaA3.MLPK*	*BnaC3.MLPK*	*BnaA4.MLPK*	*BnaC4.MLPK*
*BnaA3.MLPK*	-	98.47%	74.88%	74.88%
*BnaC3.MLPK*	98.56%	-	75.60%	75.60%
*BnaA4.MLPK*	84.73%	84.82%	-	99.76%
*BnaC4.MLPK*	84.82%	84.90%	98.09%	-

The percentages on below the diagonal line indicate CDS homology of *MLPKs*. The percentages on above the diagonal line indicate protein homology of *MLPKs*.

**Table 2:** Sequence alignment of the *MLPK* transcripts in *Brassica* species.

Transcripts	*BrMLPKf1*	*BrMLPKf2*	*BrMLPKn*	*BoMLPKf1*	*BoMLPKf2*	*BoMLPKn*
*BnaA3.MLPK*	99.83%	99.32%	81.34%	98.64%	93.29%	84.82%
*BnaC3.MLPK*	98.56%	98.05%	83.55%	98.90%	94.14%	84.90%
*BnaA4.MLPK*	85.93%	84.02%	91.77%	86.01%	80.03%	98.09%
*BnaC4.MLPK*	86.01%	84.10%	91.69%	86.09%	80.03%	100.00%

**Table 3:** Percentages of mutated plants at the T_0_ generation by single-gene targeted sgRNAs.

Target Gene	sgRNA	Number of Plants Examined	Number of Plants with Mutations	Mutation Rate (%)	Homozygous Mutations	
					Number	Rate (%)
*BnaA3.MLPK*	sgRNA1	6	6	100.0	1	0.17
	sgRNA2		6	100.0	0	0
*BnaC3.MLPK*	sgRNA1	6	6	100.0	0	0
	sgRNA2		6	100.0	0	0
*BnaA4.MLPK*	sgRNA1	6	5	83.3	0	0
	sgRNA2		5	83.3	0	0
*BnaC4.MLPK*	sgRNA1	6	6	100.0	0	0
	sgRNA2		4	66.7	0	0

**Table 4:** Genotypes of T_0_ transgenic plants.

Target Gene	Sites	Number of Examined Plants	Genotype				
			Homozygote	Heterozygote	Bi-allele	Chimera	WT
*BnaA3.MLPK*	sgRNA1	6	1 (16.7%)		3 (50.0%)	2 (33.3%)	
	sgRNA2	6			3 (50.0%)	2 (33.3%)	
*BnaC3.MLPK*	sgRNA1	6			2 (33.3%)	4 (66.7%)	
	sgRNA2	6			4 (66.7%)	2 (33.3%)	
*BnaA4.MLPK*	sgRNA1	6	1 (16.7%)	1 (16.7%)	1 (16.7%)	2 (33.3%)	1 (16.7%)
	sgRNA2	6	1 (16.7%)	1 (16.7%)	3 (50.0%)		1 (16.7%)
*BnaC4.MLPK*	sgRNA1	6	1 (16.7%)	2 (16.7%)	3 (50.0%)		
	sgRNA2	6	1 (16.7%)	1 (16.7%)	2 (33.3%)		2 (33.3%)
Total		48	5 (10.4%)	5 (10.4%)	21 (43.8%)	12 (25.0%)	4 (8.3%)

Homozygote: the two alleles have the same mutation; Bi-allele: the two alleles have different mutations; Heterozygote: only one allele is mutated; Chimera: more than two different mutations exist; WT: wild type, no mutation.

## Supplementary Materials

The following are available online at https://www.mdpi.com/1422-0067/20/13/3303/s1, Figure S1. RNA-silencing constructs used in this study; Figure S2. Information of CRISPR/Cas9 construct of editing *BnMLPK*; Figure S3. The editing information of *BnMLPK* in T_0_ generation mutants; Figure S4. The editing information of BnaMLPKs in T0 generation mutants; Table S1. The gDNA and CDS sequence of *BnMLPK*; Table S2. Primers used in the study.
