# Supplementary material for: Functional Analysis of M-Locus Protein Kinase Revealed a Novel Regulatory Mechanism of Self-Incompatibility in Brassica napus L
Source: Int J Mol Sci. 2019 Jul 5;20(13):3303. doi: 10.3390/ijms20133303 (PMC6651594; doi:10.3390/ijms20133303)
Supplement: Supplementary file 1 [file ijms-20-03303-s001.zip › Supplementary/Supplementary Figures.docx]

**Functional analysis of M-locus protein kinase revealed a novel regulatory mechanism of self-incompatibility in *Brassica napus L***

Fang Chen,^1, †^ Yong Yang,^1, †^ Bing Li, ^1^ Zhiquan Liu, ^1^ Fawad Khan, ^1^ Tong Zhang,^2^ Guilong Zhou, ^1^ Jinxing Tu, ^1^ Jinxiong Shen, ^1^ Bin Yi, ^1^ Tingdong Fu, ^1^ Cheng Dai, ^1^ * and Chaozhi Ma^1^ *

^1^National Key Laboratory of Crop Genetic Improvement, National Center of Rapeseed Improvement in Wuhan, Huazhong Agricultural University, Wuhan 430070, China

^2^Key Laboratory of Horticultural Plant Biology, Ministry of Education, Huazhong Agricultural University, Wuhan 430070, China

[Fangchen321cf@163.com](mailto:Fangchen321cf@163.com) (F.C.); yongyang218@163.com (Y.Y.); blihzau@163.com (B.L.);

lzq0826@163.com (Z.L.); fawadkhan@webmail.hzau.edu.cn (F.K.); tongtong_1024@webmail.hzau.edu.cn (T.Z.); zhouguilong@webmail.hzau.edu.cn (G.Z.); tujx@mail.hzau.edu.cn (J.T.); jxshen@mail.hzau.edu.cn (J.S.); [yibin@mail.hzau.edu.cn](mailto:yibin@mail.hzau.edu.cn) (B.Y.); futing@mail.hzau.edu.cn (T.F.)

^†^These authors have contributed equally to this work.

*****To whom correspondence should be addressed.

[cdai@mail.hzau.edu.cn](mailto:cdai@mail.hzau.edu.cn) (C.D.); [yuanbeauty@mail.hzau.edu.cn](mailto:yuanbeauty@mail.hzau.edu.cn) (C.M.); Tel.: +86-(027)-8728-1676

**Table S1** Primers used in the study.

**Table S2** The gDNA and CDS sequence of *BnaMLPKs*.


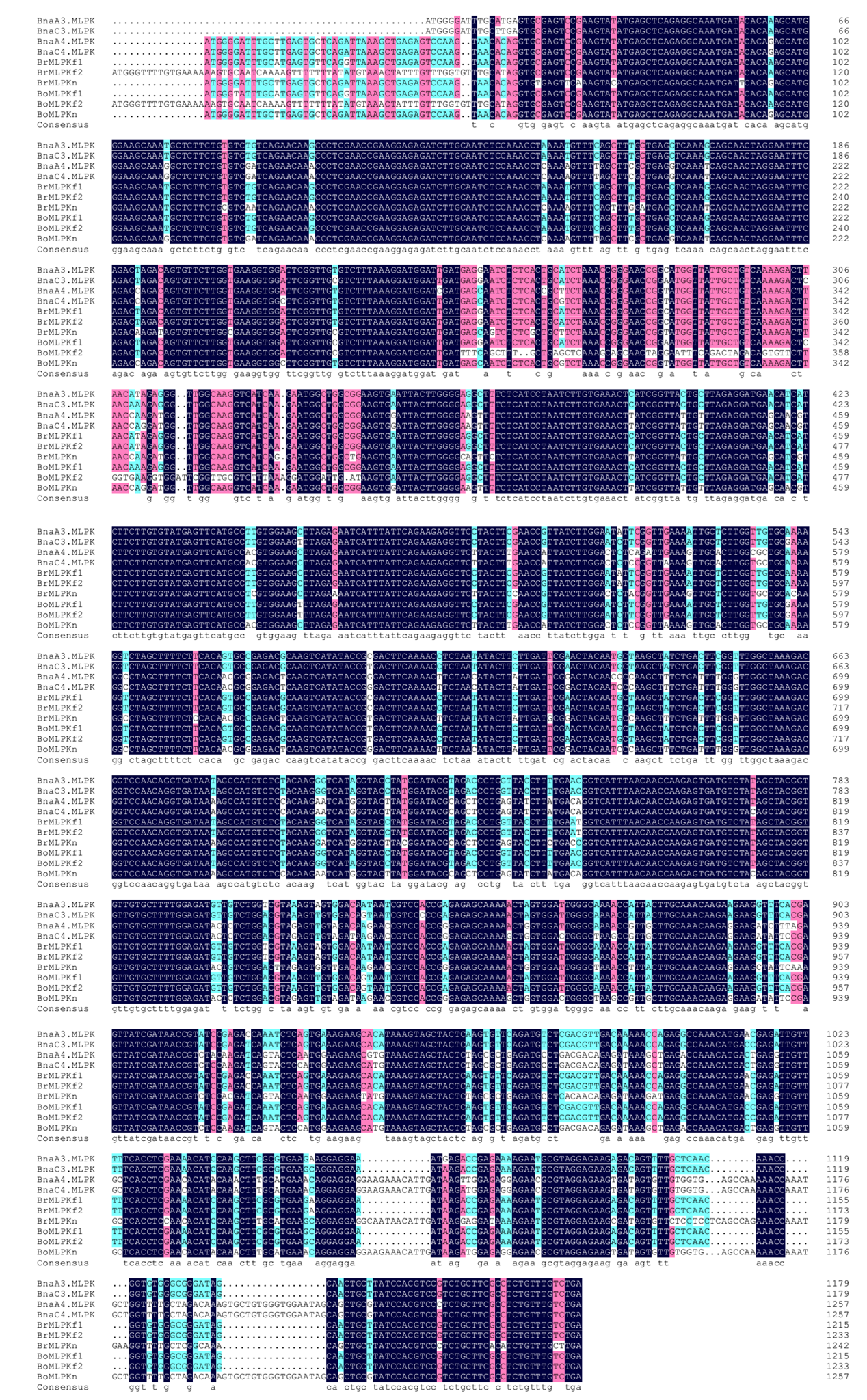


**Figure S1.** Sequential comparison of *MLPKs* CDS in *Brassica* species.


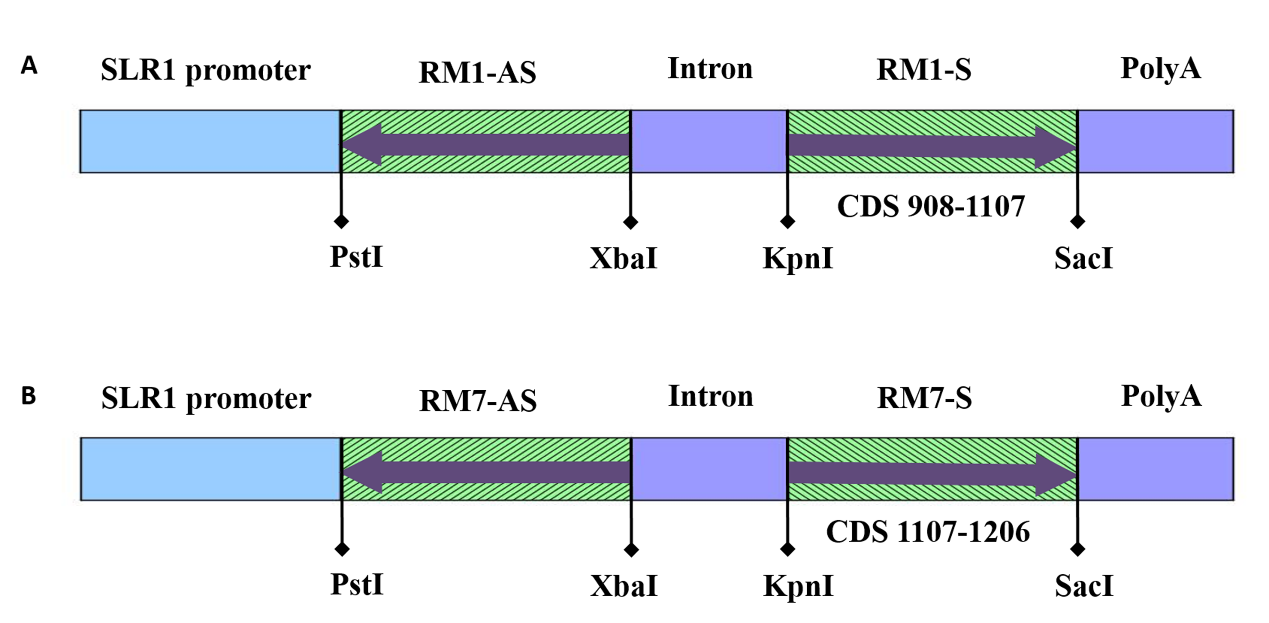


**Figure S2.** RNA-silencing constructs used in this study. The hpRNAi construct was reformed from pCAMBIA2300 vector. All constructs are driven by the stigmas specific *SLR1* promoter and stopped by PolyA. Restriction enzyme cutting site used in the study is PstI, XbaI, KpnI and SacI as indicated. **(A)** The *SLR1:RM1* hpRNAi construct contains *BnaA3.MLPK/BnaC3.MLPK* (CDS 908-1107). **(B)** The *SLR1:RM7* hpRNAi construct contains *BnaA4.MLPK/BnaC4.MLPK* (CDS 1107-1206).


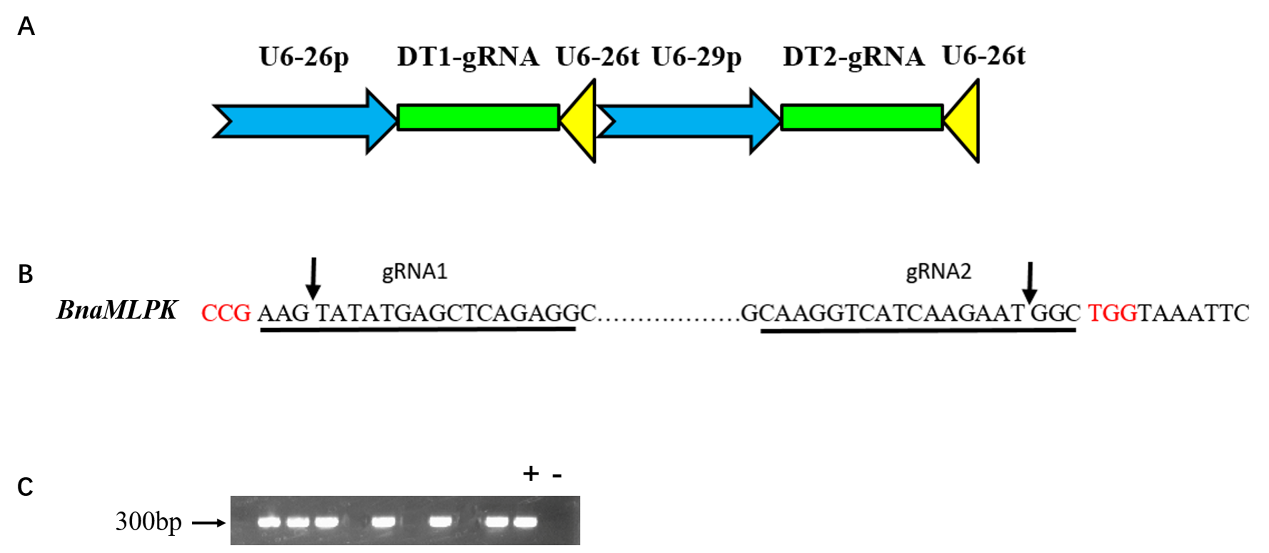


**Figure S3.** Information of CRISPR/Cas9 construct of editing *BnaMLPK*. **(A)** The two gRNA-expressing modules for *BnaMLPK*. Detailed information are previously reported [1]. **(B)** Sequences of the two sgRNA. The gRNA paired region is labeled with underline, and the PAM region is shown in red color letters. **(C)** Detecting the positive plants with Cas9-F/Cas9-R primers. The number at the start indicates the size of bands. The positive (+) and negative (−) control was marked, respectively.

**
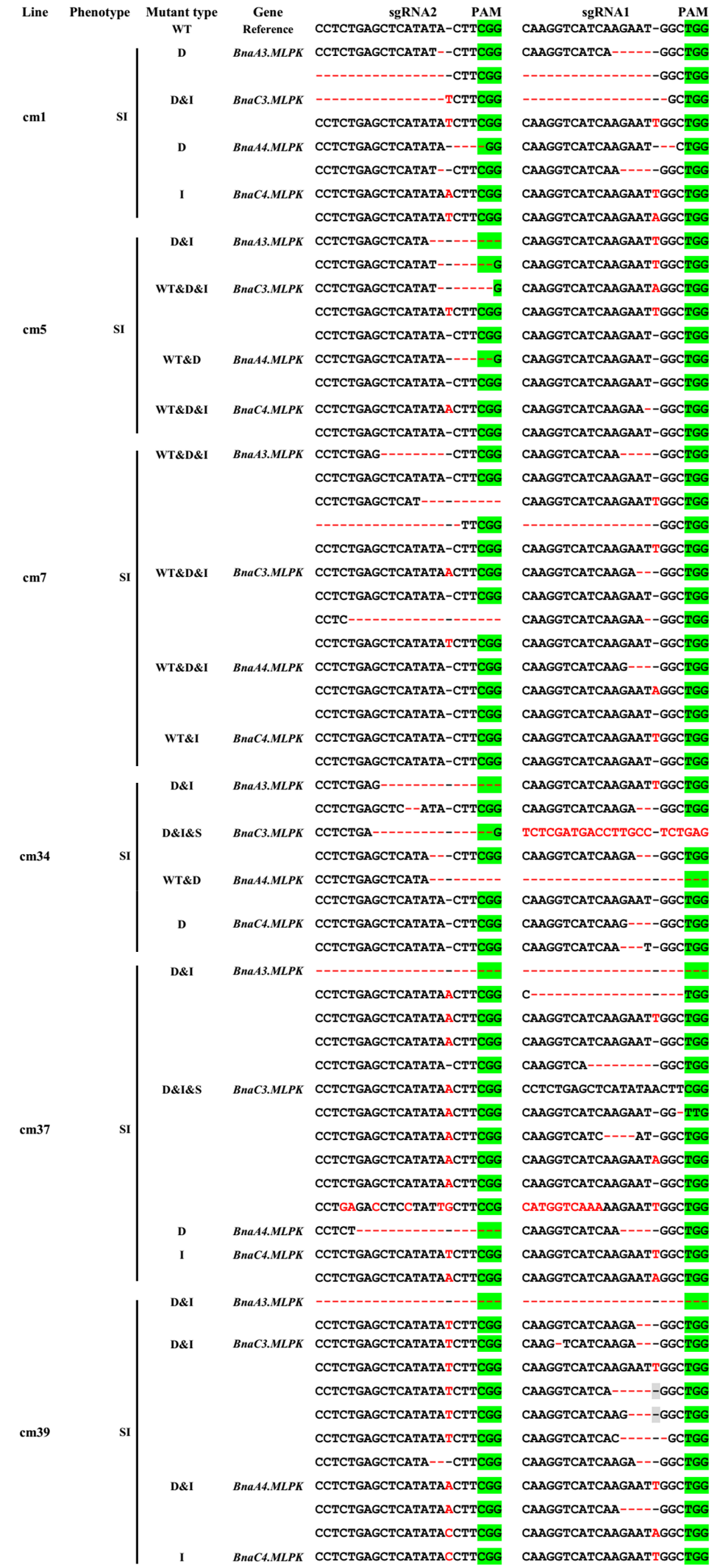
**

**Figure S4.** The editing information of *BnaMLPKs* in T_0_ generation mutants. Sequential analysis of the six T_0_ generation lines (named cm1, cm5, cm7, cm34, cm37 and cm39, respectively). The DNA was extracted from the leaves. Representative sequences of mutated *MLPK* is aligned with that of the reference gene. The PAM region is marked by green. The two sgRNA are sgRNA1 and sgRNA2, respectively. The inconsistent region indicates it is edited and labeled by red color. SI: self-incompatibility; SC: self-compatibility; D: deletion; I: insertion; S: substitution; WT: wild type ‘S-70’ sequence.
