# Supplementary material for: Functional Analysis of M-Locus Protein Kinase Revealed a Novel Regulatory Mechanism of Self-Incompatibility in Brassica napus L
Source: Int J Mol Sci. 2019 Jul 5;20(13):3303. doi: 10.3390/ijms20133303 (PMC6651594; doi:10.3390/ijms20133303)
Supplement: Supplementary file 1 [file ijms-20-03303-s001.zip › Supplementary/Supplementary Tables/Table. S1.docx]

**>*BnaA3.MLPK* (*BnaA03g22010D*) gDNA sequence**

ATGGGGATTTGCATGAGTGTTCAGGTTAAAGCTGAGAGTCCAAGTAACACAGGTACTACAAGGCTCAAGCTTTCAAATTTCATATATAGAATAACGACTACTCCTACAAATGTTTATTTGTAAACAAATATTTAATTTTGAGGTTTTAAGAGTTTGAATTTATTTTGGTCGGCAATAGAGTAGGGTCTAAACTTTAGGGTTTGTTGTTCATTTATTTTTTACGTATTTTTTTCAATTCGAGGTTAGGCTTTCCGGGACTTAAGTCACTTAACTTAAAAGTTTGGTTTCTAACGCTAAAAATAATTAGTTTTTTTTTTATATTTCAAAAAAACAAATTAATGATTAGGTTTTCGTAAACTGTCTTTTTGGGTGTTTGGCTAATTCACGGCTCATTTTAGTTGCCAGAGGAAAAGACAAGCAAGAAAATTCAGAATCTTTACATTTGTCTGATCTCTTAATTACAACAAAACCATTTGTACTAAATTTCGGATAAATGGGTTTTGTGAAAAAAGTGCAATCAAAAGTTTTTTTATATGTAAACTATTTGTTTGGTGTTTGCATAGGTGCGAGTCCGAAGTATATGAGCTCAGAGGCAAATGATACACAAAGCATGGGAAGCAAATGCTCTTCTGTGTCTGTCAGAACAAGCCCTCGAACCGAAGGAGAGATCTTGCAATCTCCAAACCTAAAATGTTTCAGCTTTGCTGAGCTCAAAGCAGCAACTAGGAATTTCAGACTAGACAGTGTTCTTGGTGAAGGTGGATTCGGTTGTGTCTTTAAAGGATGGATTGATGAGGAATCTCTCACTGCATCTAAACCGGGAACCGGCATGGTTATTGCTGTCAAAAGACTTAACATAGAGGGTTGGCAAGGTCATCAAGAATGGCTGGTAGATTCGAAAAGCTAACTTCAATCTTTTGATCACTTATATTGAAGAAAAAAAAAACTTATATTGAAGATTGTTTGTTTATGATTTTCGTTGATTAATGCTGCTGCAGGCGGAAGTGAATTACTTGGGGAGGCTTTCTCATCCTAATCTTGTGAAACTCATCGGTTACTGCTTAGAGGATGAACATCATCTTCTTGTGTATGAGTTCATGCCTTGTGGAAGCTTAGAGAATCATTTATTCAGAAGTATTGTCTTCTCTTCTCTTGTTCATTCATCTGGTAACTCGCAATGTTATTCAATCTTGTTTCTTGCTGTTACAGGAGGTTCCTACTTCGAACCGTTATCTTGGAATATTCGGTTGAAAATTGCTCTTGGTTGTGCAAAAGGTCTAGCTTTTCTTCACAGTGCCGAGACGCAAGTCATATACCGCGACTTCAAAACCTCTAATATACTTCTTGATTCGGTACTTCCATCTTTTGATATAAATGGAAAATAGCTGTCATAATTAATAAGATTTTATGTTTGAGAAAAATCTCATTGACATGATCACAGAACTACAATGCTAAGCTATCTGACTTCGGTTTGGCTAAAGACGGTCCAACAGGTGATAATAGCCATGTCTCTACAAGGGTCATAGGTACCTATGGATACGTAGACCCTGGTTACCTTTTGAACGGTTAGCCTCTCAAAAGTCTTTTTTTTTTCTTTATGTGATTTGATTTACTTCTTTATGATAACCACTAACTCTTTCACTTAGGTCATTTAACAACCAAGAGTGATGTCTATAGCTACGGTGTTGTGCTTTTGGAGATGTTGTCTGGTCGTAAAGTAGTGGACAATAATCGTCCACCGAGAGAGCAAAAACTAGTGGATTGGGCAAAACCATTACTTGCAAACAAGAAGAAGGTTTCACGAGTTATCGATAACCGTATCCGAGACCAAATCTCAGTGAAAGAAGCACATAAAGTAGCTACTCAAGTGTTCAGATGTCTCGACGTTGACAAAAACCAGAGGCCAAACATGAACGAGATTGTTTTTCACCTCGAAAACATCCAAGCTTCGCGTGAAGAAGGAGGAAATGAGACCGAGAAAAGAATGCGTAGGAGAAGAGACAGTTTTGCTCAACAAACCGGTGTGGGCGGGATAGCAACTGCTTATCCACGTCCGTCTGCTTCGCCTCTGTTTGTCTGA

**>*BnaA3.MLPKf1* CDS sequence**

ATGGGGATTTGCATGAGTGCGAGTCCGAAGTATATGAGCTCAGAGGCAAATGATACACAAAGCATGGGAAGCAAATGCTCTTCTGTGTCTGTCAGAACAAGCCCTCGAACCGAAGGAGAGATCTTGCAATCTCCAAACCTAAAATGTTTCAGCTTTGCTGAGCTCAAAGCAGCAACTAGGAATTTCAGACTAGACAGTGTTCTTGGTGAAGGTGGATTCGGTTGTGTCTTTAAAGGATGGATTGATGAGGAATCTCTCACTGCATCTAAACCGGGAACCGGCATGGTTATTGCTGTCAAAAGACTTAACATAGAGGGTTGGCAAGGTCATCAAGAATGGCTGGCGGAAGTGAATTACTTGGGGAGGCTTTCTCATCCTAATCTTGTGAAACTCATCGGTTACTGCTTAGAGGATGAACATCATCTTCTTGTGTATGAGTTCATGCCTTGTGGAAGCTTAGAGAATCATTTATTCAGAAGAGGTTCCTACTTCGAACCGTTATCTTGGAATATTCGGTTGAAAATTGCTCTTGGTTGTGCAAAAGGTCTAGCTTTTCTTCACAGTGCCGAGACGCAAGTCATATACCGCGACTTCAAAACCTCTAATATACTTCTTGATTCGAACTACAATGCTAAGCTATCTGACTTCGGTTTGGCTAAAGACGGTCCAACAGGTGATAATAGCCATGTCTCTACAAGGGTCATAGGTACCTATGGATACGTAGACCCTGGTTACCTTTTGAACGGTCATTTAACAACCAAGAGTGATGTCTATAGCTACGGTGTTGTGCTTTTGGAGATGTTGTCTGGTCGTAAAGTAGTGGACAATAATCGTCCACCGAGAGAGCAAAAACTAGTGGATTGGGCAAAACCATTACTTGCAAACAAGAAGAAGGTTTCACGAGTTATCGATAACCGTATCCGAGACCAAATCTCAGTGAAAGAAGCACATAAAGTAGCTACTCAAGTGTTCAGATGTCTCGACGTTGACAAAAACCAGAGGCCAAACATGAACGAGATTGTTTTTCACCTCGAAAACATCCAAGCTTCGCGTGAAGAAGGAGGAAATGAGACCGAGAAAAGAATGCGTAGGAGAAGAGACAGTTTTGCTCAACAAACCGGTGTGGGCGGGATAGCAACTGCTTATCCACGTCCGTCTGCTTCGCCTCTGTTTGTCTGA

**>*BnaA3.MLPKf2* CDS sequence**

ATGGGTTTTGTGAAAAAAGTGCAATCAAAAGTTTTTTTATATGTAAACTATTTGTTTGGTGTTTGCATAGGTGCGAGTCCGAAGTATATGAGCTCAGAGGCAAATGATACACAAAGCATGGGAAGCAAATGCTCTTCTGTGTCTGTCAGAACAAGCCCTCGAACCGAAGGAGAGATCTTGCAATCTCCAAACCTAAAATGTTTCAGCTTTGCTGAGCTCAAAGCAGCAACTAGGAATTTCAGACTAGACAGTGTTCTTGGTGAAGGTGGATTCGGTTGTGTCTTTAAAGGATGGATTGATGAGGAATCTCTCACTGCATCTAAACCGGGAACCGGCATGGTTATTGCTGTCAAAAGACTTAACATAGAGGGTTGGCAAGGTCATCAAGAATGGCTGGCGGAAGTGAATTACTTGGGGAGGCTTTCTCATCCTAATCTTGTGAAACTCATCGGTTACTGCTTAGAGGATGAACATCATCTTCTTGTGTATGAGTTCATGCCTTGTGGAAGCTTAGAGAATCATTTATTCAGAAGAGGTTCCTACTTCGAACCGTTATCTTGGAATATTCGGTTGAAAATTGCTCTTGGTTGTGCAAAAGGTCTAGCTTTTCTTCACAGTGCCGAGACGCAAGTCATATACCGCGACTTCAAAACCTCTAATATACTTCTTGATTCGAACTACAATGCTAAGCTATCTGACTTCGGTTTGGCTAAAGACGGTCCAACAGGTGATAATAGCCATGTCTCTACAAGGGTCATAGGTACCTATGGATACGTAGACCCTGGTTACCTTTTGAACGGTCATTTAACAACCAAGAGTGATGTCTATAGCTACGGTGTTGTGCTTTTGGAGATGTTGTCTGGTCGTAAAGTAGTGGACAATAATCGTCCACCGAGAGAGCAAAAACTAGTGGATTGGGCAAAACCATTACTTGCAAACAAGAAGAAGGTTTCACGAGTTATCGATAACCGTATCCGAGACCAAATCTCAGTGAAAGAAGCACATAAAGTAGCTACTCAAGTGTTCAGATGTCTCGACGTTGACAAAAACCAGAGGCCAAACATGAACGAGATTGTTTTTCACCTCGAAAACATCCAAGCTTCGCGTGAAGAAGGAGGAAATGAGACCGAGAAAAGAATGCGTAGGAGAAGAGACAGTTTTGCTCAACAAACCGGTGTGGGCGGGATAGCAACTGCTTATCCACGTCCGTCTGCTTCGCCTCTGTTTGTCTGA

**>*BnaC3.MLPK* (*BnaC03g26350D*) gDNA sequence**

ATGGGGATTTGCATGAGTGTTCAGATTAAAGCTGAGAGTCCAAGTAACACAGGTACTACAAGGCTCAAGCTTTCAATTTTCATATATAGAATAACGACTACTCCTGCAAATGTTTATTATTTAATTTTGAGGTTTTAAGAGTTTGAATTTATTTTGGTTGGCAATAGAGTAGGGTCTAAACTTTAGGGTTTGTTGTTCATTTATTTTTTACGTATTTTTTTCAATTCGAGGTTAGGCTTTCCGGGACTTAAGTCACTTAACTTAAAAGTTTGGTTTCTAACGCTAAAAATAATTAGTTTTTTTTTTTATATTTCAAAAAACAAATTAATGATTAGGTTTTCGTAAACTGTCTTTTTGGGTGTTTGGCTAATTCACGGCTCATTTTAGTTGCCAGAGGAAAAGACAAGCAAGAAAATTCAGAATCTTTACATTTGTCTGATCTCTTAATTACAACAAAACCATTTGTACTAAATTTCGGATAAATGGGTTTTGTGAAAAAAGTGCAATCAAAAGTTTTTTTATATGTAAACTATTTGTTTGGTGTTTGCATAGGTGCGAGTCCGAAGTATATGAGCTCAGAGGCAAATGATACACAAAGCATGGGAAGCAAATGCTCTTCTGTGTCTGTCAGAACAAGCCCTCGAACCGAAGGAGAGATCTTGCAATCTCCAAACCTAAAATGTTTCAGCTTTGCTGAGCTCAAAGCAGCAACTAGGAATTTCAGACTAGACAGTGTTCTTGGTGAAGGTGGATTCGGTTGCGTCTTTAAAGGATGGATTGATGAGGAATCTCTCACTGCATCTAAACCGGGAACCGGAATGGTTATTGCTGTCAAAAGACTCAACAAAGAGGGTTGGCAAGGTCATCAAGAATGGCTGGTAGATTCGAAAAGCTAACTTCAATCTTTTGATCACTTATATTGAAGAAAAAAAAATTATATTGAAGATTGTTTGTTTATGATTTTCGTTGATTAATGCTGCTGCAGGCGGAAGTGAATTACTTGGGGAGGCTTTCTCATCCTAATCTTGTGAAACTCATCGGTTACTGCTTAGAGGATGAACATCATCTTCTTGTGTATGAGTTCATGCCTTGTGGAAGTTTAGAGAATCATTTATTCAGAAGTATTGTCTTGTCTTCTCTTGTTCATTCATCTGGTAACTCGCAATGTTATTCAATCTTGTTTCTTGCTGTTACAGGAGGTTCCTACTTCGAACCGTTATCTTGGAATCTTCGGTTGAAAATTGCTCTTGGTTGTGCGAAAGGTCTAGCTTTTCTTCACAGTGCCGAGACGCAAGTCATATACCGTGACTTCAAAACCTCTAATATACTTCTTGATTCGGTACTTCCATCTTTTGATATAATGAAAAATAGCTGTGATAATAAGATTTTATGTTTGAGAAAAATCTCATTGACATGATCACAGAACTACAATGCTAAGCTATCTGACTTCGGTTTGGCTAAAGACGGTCCAACAGGTGATAATAGCCATGTCTCTACAAGGGTCATAGGTACCTATGGATACGTAGACCCTGGTTACCTTTTGAACGGTTAGCCTCTCAAAAGTCTTTTTTTCTTTATGTGATTTGATTTACTTCTTTATGATAACCACTAACTCTTTCACTTAGGTCATTTAACAACCAAGAGTGATGTCTATAGCTACGGTGTTGTGCTTTTGGAGATGTTGTCTGGACGTAAAGTTGTGGACAGTAATCGTCCCCCGAGAGAGCAAAAACTAGTGGATTGGGCAAAACCATTACTTGCAAACAAGAAGAAGGTTTCACGAGTTATCGATAACCGTATCCGAGATCAAATCTCAGTGAAAGAAGCACATAAAGTAGCTACTCAAGTGTTCAGATGTCTCGACGTTGACAAAAACCAGAGGCCAAACATGACCGAGATTGTTTTTCACCTCGAAAACATCCAAGCTTCGCGTGAAGCAGGAGGAAATAAGACCGAGAAAAGAATGCGTAGGAGAAGAGACAGTTTTGCTCAACAAACCGGTGTGGGCGGGATAGCAACTGCTTATCCACGTCCGTCTGCTTCGCCTCTGTTTGTCTGA

**>*BnaC3.MLPK* CDS sequence**

ATGGGGATTTGCTTGAGTGCGAGTCCGAAGTATATGAGCTCAGAGGCAAATGATACACAAAGCATGGGAAGCAAATGCTCTTCTGTGTCTGTCAGAACAAGCCCTCGAACCGAAGGAGAGATCTTGCAATCTCCAAACCTAAAATGTTTCAGCTTTGCTGAGCTCAAAGCAGCAACTAGGAATTTCAGACTAGACAGTGTTCTTGGTGAAGGTGGATTCGGTTGCGTCTTTAAAGGATGGATTGATGAGGAATCTCTCACTGCATCTAAACCGGGAACCGGAATGGTTATTGCTGTCAAAAGACTCAACAAAGAGGGTTGGCAAGGTCATCAAGAATGGCTGGCGGAAGTGAATTACTTGGGGAGGCTTTCTCATCCTAATCTTGTGAAACTCATCGGTTACTGCTTAGAGGATGAACATCATCTTCTTGTGTATGAGTTCATGCCTTGTGGAAGTTTAGAGAATCATTTATTCAGAAGAGGTTCCTACTTCGAACCGTTATCTTGGAATCTTCGGTTGAAAATTGCTCTTGGTTGTGCGAAAGGTCTAGCTTTTCTTCACAGTGCCGAGACGCAAGTCATATACCGTGACTTCAAAACCTCTAATATACTTCTTGATTCGAACTACAATGCTAAGCTATCTGACTTCGGTTTGGCTAAAGACGGTCCAACAGGTGATAATAGCCATGTCTCTACAAGGGTCATAGGTACCTATGGATACGTAGACCCTGGTTACCTTTTGAACGGTCATTTAACAACCAAGAGTGATGTCTATAGCTACGGTGTTGTGCTTTTGGAGATGTTGTCTGGACGTAAAGTTGTGGACAGTAATCGTCCCCCGAGAGAGCAAAAACTAGTGGATTGGGCAAAACCATTACTTGCAAACAAGAAGAAGGTTTCACGAGTTATCGATAACCGTATCCGAGATCAAATCTCAGTGAAAGAAGCACATAAAGTAGCTACTCAAGTGTTCAGATGTCTCGACGTTGACAAAAACCAGAGGCCAAACATGACCGAGATTGTTTTTCACCTCGAAAACATCCAAGCTTCGCGTGAAGCAGGAGGAAATAAGACCGAGAAAAGAATGCGTAGGAGAAGAGACAGTTTTGCTCAACAAACCGGTGTGGGCGGGATAGCAACTGCTTATCCACGTCCGTCTGCTTCGCCTCTGTTTGTCTGA

**>*BnaA4.MLPK* (*BnaA04g16770D*) gDNA sequence**

ATGGGGATTTGCTTGAGTGCTCAGATTAAAGCTGAGAGTCCAAGTAACACAGGTACTTCAATACTCAAGCTTTCATTTACATATATACAATAACCACTACTCATGCAAATATTTAGATGTGTTTTTATAAGTTTATTTTATCAATTTAAGGTTAGGTATTTGGTAATTGAGACATAAAAAGTGACTTAACTTAAAAGTTAAAGCTTAGGTGTTTTTGACAAATCTGTCTTTTCCAACGCTAATAATTTGATTAATTCTTAAGCTCTGTCTTCTTCATAATCTTTGCACTATGTTTTGAAGAGATGGGTTTTGTCAAAAATATGAAATGTATATTTAGAAACTATTTGTTTGGTGTTTGCATAGGTGCGAGTCCGAAGTATATGAGCTCAGAGGCAAATGATACACAGAGCATGGGAAGCAAAGGCTCTTCTGTGTCGATCAGAACAAACCCTCGAACCGAAGGAGAGATCTTGCAATCTCCAAACCTCAAAAGTTTTAGCTTCGCTGAGGTCAAATCAGCAACTAGGAATTTCAGACCAGACAGTGTTCTTGGTGAAGGTGGATTCGGTTGTGTCTTTAAAGGATGGATCGATGAGCAATCTCTCACCGCTTCTAAACCGGGAACCGGTATGGTTATTGCTGTCAAAAGACTTAACCAAGATGGTTGGCAAGGTCATCAAGAATGGCTGGTAAATTCAGAATCAGATCTTTTGAAATTATATTTGAAGATTGTTTGTTTATGTTTTCTACAATGATGTTGTTATAGGCGGAAGTGGATTACTTGGGGAAGTTTTCTCATCCTAATCTTGTGAAACTTATCGGTTATTGTTTAGAGGATGAGCAACGTCTTCTTGTGTATGAGTTCATGCCACGTGGAAGCTTAGAGAATCATTTATTCAGAAGTATTGTCTTCTCTTCTCTTGTATATATTCCTTTAGTAACCTCTAAATGTTTCTTGAGTTTTTGAATCTTGTTCCTTGATGTTTTTGCAGGAGGTTCTTACTTTGAACCATTATCTTGGACTCTCAGATTGAAAGTTGCACTTGGCGCTGCAAAAGGCCTAGCTTTTCTTCACAACGCGGAGACTCAAGTCATATACCGGGACTTCAAAACTTCTAACATACTTATTGATTCGGTACTTCAGCCTTCTTCCTTCATTTTGATATGATGAAAAATGCTGTGTGAAAGTTTGATATACGTATACTTTGGTCATCTCTTTGACATGATCACAGGACTACAACCCCAAGCTTTCTGATTTTGGGTTGGCTAAAGACGGTCCAACAGGTGATAAAAGCCATGTCTCCACAAGAATCATGGGTACTTATGGATACGCAGCTCCTGAGTATCTTATGACAGGTTAGTCTCTCACACATTTTATGTGACTTTTTATCAGTGTTCTAAAAATTGGTCTAGATGGCGCCTAAGCGGCAAATCAGTCATATCCGCGATTTTCTGATTTATGCAACTCAAATGGATAACCGAGTTAAGCAATAATATTAGGCACATATTAGTTTTTATAATTCATCAAAAAAAAATTTCCATTTAACTTTTTTTGTATGTATAGCTTTATATAATTCATCAAAATAATTTTATAGTTAAACCCAAATACTAAAATATCTAATATAAGTGTAAATATATAAAAATAAATCAATAATTCCATATTGTAGTTGCTAGTTTTATATGAAGCGACTAGTTGCTGCCTAGCAATTATTTGAACACTGCTTTTTATGATGACCACTGACTGTTTCACTTTCCTTAGGTCATTTAACAACCAAGAGTGATGTCTATAGCTACGGTGTTGTGCTTTTGGAGATACTCTCTGGACGTAGAGTTGTAGACAAGAACCGTCCACCGGGAGAGCAAAAACTGGTGGATTGGGCAAAACCGTTGCTTGCAAACAAGAGGAAGATCTTTAGAGTTATCGATAACCGTCTACAAGATCAGTACTCAATGGAAGAAGCGTGTAAAGTAGCTACTCTAGCGCTGAGATGCCTGACGACAGAGATAAAGCTGAGACCAAACATGACTGAGGTTGTTGCTCACCTCGAACACATACAAACTTTGCATGAAACAGGAGGAGGAAGAAACATTGATAAGTTGGAGAGGAGAACGCGTAGGAGAAGTGATAGTGTTGTGGTGAGCCAAAAACCAAATGCTGGTTTTGCTAGACAAAGTGCTGTGGGTGGAATAGCAGCTGCGTATCCACGTCCCTCTGCTTCGCCTCTGTTTGTCTGA

**>*BnaA4.MLPK* CDS sequence**

ATGGGGATTTGCTTGAGTGCTCAGATTAAAGCTGAGAGTCCAAGTAACACAGGTGCGAGTCCGAAGTATATGAGCTCAGAGGCAAATGATACACAGAGCATGGGAAGCAAAGGCTCTTCTGTGTCGATCAGAACAAACCCTCGAACCGAAGGAGAGATCTTGCAATCTCCAAACCTCAAAAGTTTTAGCTTCGCTGAGGTCAAATCAGCAACTAGGAATTTCAGACCAGACAGTGTTCTTGGTGAAGGTGGATTCGGTTGTGTCTTTAAAGGATGGATCGATGAGCAATCTCTCACCGCTTCTAAACCGGGAACCGGTATGGTTATTGCTGTCAAAAGACTTAACCAAGATGGTTGGCAAGGTCATCAAGAATGGCTGGCGGAAGTGGATTACTTGGGGAAGTTTTCTCATCCTAATCTTGTGAAACTTATCGGTTATTGTTTAGAGGATGAGCAACGTCTTCTTGTGTATGAGTTCATGCCACGTGGAAGCTTAGAGAATCATTTATTCAGAAGAGGTTCTTACTTTGAACCATTATCTTGGACTCTCAGATTGAAAGTTGCACTTGGCGCTGCAAAAGGCCTAGCTTTTCTTCACAACGCGGAGACTCAAGTCATATACCGGGACTTCAAAACTTCTAACATACTTATTGATTCGGACTACAACCCCAAGCTTTCTGATTTTGGGTTGGCTAAAGACGGTCCAACAGGTGATAAAAGCCATGTCTCCACAAGAATCATGGGTACTTATGGATACGCAGCTCCTGAGTATCTTATGACAGGTCATTTAACAACCAAGAGTGATGTCTATAGCTACGGTGTTGTGCTTTTGGAGATACTCTCTGGACGTAGAGTTGTAGACAAGAACCGTCCACCGGGAGAGCAAAAACTGGTGGATTGGGCAAAACCGTTGCTTGCAAACAAGAGGAAGATCTTTAGAGTTATCGATAACCGTCTACAAGATCAGTACTCAATGGAAGAAGCGTGTAAAGTAGCTACTCTAGCGCTGAGATGCCTGACGACAGAGATAAAGCTGAGACCAAACATGACTGAGGTTGTTGCTCACCTCGAACACATACAAACTTTGCATGAAACAGGAGGAGGAAGAAACATTGATAAGTTGGAGAGGAGAACGCGTAGGAGAAGTGATAGTGTTGTGGTGAGCCAAAAACCAAATGCTGGTTTTGCTAGACAAAGTGCTGTGGGTGGAATAGCAGCTGCGTATCCACGTCCCTCTGCTTCGCCTCTGTTTGTCTGA

**>*BnaC4.MLPK* (*BnaC04g40150D*) gDNA sequence**

ATGGGGATTTGCTTGAGTGCTCAGATTAAAGCTGAGAGTCCAAGTAACACAGGTACTTCAATGCTCAAGCTTTCATTTACATATATACAATAACCACTACTCATGCAAATATTTAGATTTGTTACTTATGTGTTTTTATAAGTTTATTTTATCAATTCGAGGTTAGGTTTTTGGTGGTTGAGACATATCAAGGGACTTAACTTAAAAGTTAAGCTTAGTGTTTTTGACAAATCTGTCTTTTCTAACGCTAATAATTGATTAATTCTTCTAAAGCTCTGTCTTCTTCATAATCTCTGCACTATGTTTTGTTGTCAAAAATGTGAAATATAAAGTTTTCTTATATACAAACCATTTGTTTGGTGTTTGCATAGGT

GCGAGTCCGAAGTATATGAGCTCAGAGGCAAATGATACACAGAGCATGGGAAGCAAAGGCTCTTCTGTGTCGATCAGAACAAACCCTCGAACCGAAGGAGAGATCTTGCAATCTCCAAACCTCAAAAGTTTTAGCTTCGCTGAGGTCAAATCAGCAACTAGGAATTTCAGACCAGACAGTGTTCTTGGTGAAGGTGGCTTCGGTTGTGTCTTTAAAGGATGGATTGATGAGCAATCTCTCACTGCGTCTAAACCGGGAACCGGTATGGTTATTGCTGTCAAAAGACTTAACCAGGATGGTTGGCAAGGTCATCAAGAATGGCTGGTAAAATTCAGAATCAGATCTTTTGAAATTATATTTGAAGATTGTTTGTTTATGTTTTTCTACAATGATGTTATAGGCGGAAGTGGATTACTTGGGGAAGTTTTCTCATCCTAATCTTGTGAAACTTATCGGTTATTGTTTAGAGGATGAGCAACGTCTTCTTGTGTATGAGTTCATGCCACGTGGAAGCTTAGAGAATCATTTATTCAGAAGTATTGTCTTCTCTTCTCTTGTCTATATTCCTCTCGTAACTCTAAATGTTTCTTGAGTATTGAATCTTGTGTTCTTGGTGTTGTTGCAGGAGGTTCTTACTTTGAACCATTATCTTGGACTCTCCGGTTAAAAGTTGCACTTGGTGCTGCAAAAGGCCTAGCTTTTCTTCACAACGCGGAGACTCAAGTCATATACCGGGACTTCAAAACTTCTAACATACTTATTGATTCGGTACTTCAGCCTTCTTCCTTCATTTTGATATGCTGAAAAATGCTGTGTGAAAGTTTGATATACGTATACTTTGGTCACCTCTTTGACATGATCACAGGACTACAATCCCAAGCTTTCTGATTTTGGGTTGGCTAAAGACGGTCCAACAGGTGATAAAAGCCATGTCTCCACAAGAATCATGGGTACTTATGGATACGCAGCTCCTGAGTATCTTATGACAGGTTAGTCTCTCAAACATCTTTTAAGCGACTTTTTATTAGTGTTCTAAAAATCGGTTTAGAAAGCGCCTAAACGGCAAATCGGTCATATCCGTAACAATTTTCTTAAAATCCGATATATACAACTCAGATTGATTCTAAAATTGGTGGAGATTGATCTAAATTGATCTGCATATGTTAAAATAAACAATAATATTAGGGCAACAAATTTGTCTAGTTTATTTTTTTGTATGCATAATTTTATGATTCATTAAAAGTTTTATGAGTGAATCCAAAAACTAAAATATTTAATGTAAATGTAAAATATGAAAAAAAATTAATGATTTGTTAACGCCTAACTCCGAATAATCTGCATAGTCGTTAGTACTATATGAAGTGTCTGTCTAGTTACCGTCTAACAATTTCTTGCACATTGTTTTTTAAGATGACCACTGACTGTTTCACTTTCCTTAGGTCATTTAACAACCAAGAGTGATGTCTACAGCTACGGTGTTGTGCTTTTGGAGATACTCTCTGGACGTAGAGTTGTAGATAAGAACCGTCCACCGGGAGAGCAAAAGCTGGTGGACTGGGCTAAGCCGTTGCTTGCAAACAAGAGGAAGATATTCCGAGTTATCGATAACCGTCTCCAAGATCAGTACTCCATGGAAGAAGCATGTAAAGTAGCTACTCTAGCGCTGAGATGCCTGACGACAGAGATAAAGCTGAGACCAAACATGACTGAGGTTGTTGCTCACCTCGAACACATACAAACTTTGCATGAAACAGGAGGAGGAAGAAACATTGATAAGATGGAGAGGAGAACGCGTAGGAGAAGTGATAGTGTTGTGGTGAGCCAAAAACCAAATGCTGGTTTTGCTAGACAAAGTGCTGTGGGTGGAATAGCAGCTGCGTATCCACGTCCGTCTGCTTCGCCTCTGTTTGTCTGA

**>*BnaC4.MLPK* CDS sequence**

ATGGGGATTTGCTTGAGTGCTCAGATTAAAGCTGAGAGTCCAAGTAACACAGGTGCGAGTCCGAAGTATATGAGCTCAGAGGCAAATGATACACAGAGCATGGGAAGCAAAGGCTCTTCTGTGTCGATCAGAACAAACCCTCGAACCGAAGGAGAGATCTTGCAATCTCCAAACCTCAAAAGTTTTAGCTTCGCTGAGGTCAAATCAGCAACTAGGAATTTCAGACCAGACAGTGTTCTTGGTGAAGGTGGCTTCGGTTGTGTCTTTAAAGGATGGATTGATGAGCAATCTCTCACTGCGTCTAAACCGGGAACCGGTATGGTTATTGCTGTCAAAAGACTTAACCAGGATGGTTGGCAAGGTCATCAAGAATGGCTGGCGGAAGTGGATTACTTGGGGAAGTTTTCTCATCCTAATCTTGTGAAACTTATCGGTTATTGTTTAGAGGATGAGCAACGTCTTCTTGTGTATGAGTTCATGCCACGTGGAAGCTTAGAGAATCATTTATTCAGAAGAGGTTCTTACTTTGAACCATTATCTTGGACTCTCCGGTTAAAAGTTGCACTTGGTGCTGCAAAAGGCCTAGCTTTTCTTCACAACGCGGAGACTCAAGTCATATACCGGGACTTCAAAACTTCTAACATACTTATTGATTCGGACTACAATCCCAAGCTTTCTGATTTTGGGTTGGCTAAAGACGGTCCAACAGGTGATAAAAGCCATGTCTCCACAAGAATCATGGGTACTTATGGATACGCAGCTCCTGAGTATCTTATGACAGGTCATTTAACAACCAAGAGTGATGTCTACAGCTACGGTGTTGTGCTTTTGGAGATACTCTCTGGACGTAGAGTTGTAGATAAGAACCGTCCACCGGGAGAGCAAAAGCTGGTGGACTGGGCTAAGCCGTTGCTTGCAAACAAGAGGAAGATATTCCGAGTTATCGATAACCGTCTCCAAGATCAGTACTCCATGGAAGAAGCATGTAAAGTAGCTACTCTAGCGCTGAGATGCCTGACGACAGAGATAAAGCTGAGACCAAACATGACTGAGGTTGTTGCTCACCTCGAACACATACAAACTTTGCATGAAACAGGAGGAGGAAGAAACATTGATAAGATGGAGAGGAGAACGCGTAGGAGAAGTGATAGTGTTGTGGTGAGCCAAAAACCAAATGCTGGTTTTGCTAGACAAAGTGCTGTGGGTGGAATAGCAGCTGCGTATCCACGTCCGTCTGCTTCGCCTCTGTTTGTCTGA
